# 

**Published:** 1890-11

**Authors:** 


### THE PRESENT NEED OF THE JOURNAL.

As we are soon to enter a new year, this becomes the period when all subscriptions should be renewed. The support of the various and considerable expenses connected with the publication of a journal depends upon the degree to which the appeal for subscriptions is responded to. If this be indifferent, the effort to extend the work is hampered. In case the reply is a generous one, there is scarcely a limit to what may be done in the direction of improvement. We have been faithfully endeavoring to satisfy the wants of our readers, and feel we are entitled to generous support. We therefore, with earnestness, ask each present subscriber to renew, and also desire all old subscribers, who for any reason have suspended their subscriptions, to again forward their names and money.

We also beg each subscriber to interest himself to solicit others to join him in aiding this endeavor, and we further trust each member of the company, wherever situated, will help in the effort to greatly increase circulation for the coming year.

We respectfully call attention to the price and terms on the first page of the cover, and also to the red folio in this and the next issue.

It may not be improper here to state that this journal is under the auspices of many leading dentists of various parts of the country, and is an outcome of their desire that the profession may have a mouth-piece for the full expression of its wants, and which may become, if properly supported, superior to any dental journal heretofore known. The members of the Publication Company under no circumstances can have any share of the pecuniary results, these being devoted to the production and improvement of the journal. As the journal, therefore, exists solely for the advancement of the profession through the record of facts and the instruction given, it appears to be the duty of every dentist to aid a cause which either directly or indirectly is to his interest, and which also is conducive to the good of the whole body of our fraternity. *

